# Transanal hemorrhoidal dearterialization (THD) for hemorrhoidal disease: An Italian single-institution 5-year experience analysis and updated literature review

**DOI:** 10.3389/fsurg.2022.1088546

**Published:** 2022-12-21

**Authors:** Luigi Verre, Gaetano Gallo, Giulia Grassi, Edoardo Bussolin, Ludovico Carbone, Gianmario Edoardo Poto, Osvaldo Carpineto Samorani, Luigi Marano, Daniele Marrelli, Franco Roviello

**Affiliations:** ^1^ Department of Medicine, Surgery and Neurosciences, Unit of General Surgery and Surgical Oncology, University of Siena, Siena, Italy; ^2^ Department of Surgical Sciences, La Sapienza University of Roma, Roma, Italy

**Keywords:** transanal hemorrhoidal dearterialization, hemorrhoids, recurrence, quality of life, outcomes

## Abstract

**Background:**

Hemorrhoidal disease is a highly prevalent, chronic disorder that usually compromise patients' quality of life. Despite recent advances in pharmacologic and surgical therapeutic options, a clear treatment “gold standard” is lacking. Our aim is to analyze the outcomes following Transanal Hemorrhoidal Dearterialization (THD) procedure.

**Methods:**

Patients who failed conservative treatment and underwent THD Doppler between 2017 and 2021 were enrolled. Follow-up interviews (consisting of clinical examination, Visual Analog Scale for pain—VAS, Vaizey incontinence score, Hemorrhoid Severity Score) were administered 1 week, 2 weeks, 1 month and 6 months after surgery.

**Results:**

Forty-seven out of 75 patients were male, and the mean age was 50 (± 17.9) years. Hemorrhoids were classified as Goligher's degree II in 25 cases, III in 40 and IV, simple irreducible without ischemic changes, in 10. The mean operative time was 35 (28–60) minutes, and most procedures were performed with epidural anesthesia (80%). No intraoperative complications occurred, and 73 patients (97.3%) were discharged within post-operative day 1. Early post-operative pain and bleeding occurred in 37.3% and 8% of patients, respectively. No patients experienced anal incontinence and severe symptoms at 6 months after surgery. The overall success rate was 97.3%.

**Conclusions:**

THD is safe and effective in hemorrhoidal disease at degree II if bleeding, III, and IV without ischemic changes, both as a first intervention and on recurrence. Physician and patient need to understand each other's expectations, weight the risks and benefits, and customize the treatment.

## Introduction

Hemorrhoidal disease (HD) is a prevalent and debated proctologic condition (1). According to the severity of the disease (2), different treatment options, ranging from dietary-lifestyle measures to surgical treatment, have been proposed (3–5). However, the commonly adopted Goligher Classification (2) does not comprehensively consider the etiopathogenesis, the symptoms of the disease, their influence on the quality of life (QoL) (6), and need to be supplemented with clinical characteristics.

In the last few years, non-excisional surgical treatments have gained increasing popularity because they allowed to reduce most patients’ discomforts, such as post-operative pain and recovery of working independence (7), with the advantage of keeping in place a physiologically useful tissue both for the defecation and continence. The Transanal Hemorrhoidal Dearterialization (THD), firstly described in 1995 (8), represents a valid choice in patients with II to IV degree HD (9), despite possible recurrences, whose risk is higher the greater the severity of the disease (10, 11).

Several variants of THD procedure have been described in recent years: doppler-guided hemorrhoidal artery ligation (DGHAL) (12), targeted mucopexy (13) and Anolift (14). The DGHAL allows the surgeon to identify and ligate the terminal branches of the superior rectal artery that feed the hemorrhoidal plexus. Frequently, the surgical indications are also expanded to the prolapses of hemorrhoidal tissue by carrying out standard mucopexy (12, 13) or recent Anolift procedure, conceived to overcome the inadequacy of the needle shape (14). However, no technical variant has been shown to be superior to the other while the surgeon's experience can improve the outcomes (5, 10, 15).

In 2018, the American Society of Colon and Rectal Surgeons (ASCRS) clinical practice guidelines listed DGHAL with mucopexy among surgical treatments for hemorrhoids (16). A consensus statement from the Italian Society of Colorectal Surgery (SICCR) (9, 17), aiming at establishing an evidence-based approach to HD, described THD and DGHAL techniques as associated with lower postoperative pain and faster recovery than excisional hemorrhoidectomy (i.e., Milligan-Morgan and Ferguson procedures, or radiofrequency hemorrhoidectomy), but carries higher recurrence rates [*Level of evidence 1, Grade of recommendation A* (18)]. The current recurrence rate ranged between 3% and 20%, with 4.1–17.8% of patients required reoperation (13).

The aim of the present observational study is to show the outcomes of the last 75 THDs performed in our center. We provide a critical review of the literature, giving evidence-based recommendations to improve patients' postoperative QoL.

## Materials and methods

### Study design

Between January 2017 and December 2021, a total of 75 patients underwent THD for HD in our center. All procedures were performed by the same colorectal surgeon (LV) and recorded in a prospectively maintained database. Demographic data, the type and severity of symptoms, anal continence status and procedural details including perioperative (comorbidities) and intraoperative data, length of hospital stay, readmission rate, and other short-term outcomes were analyzed.

The results of this study were reported as established by the Strengthening the reporting of observational studies in epidemiology (STROBE) statement for cohort studies (19).

The severity of disease was evaluated with a complete proctological examination, including both digital rectal examination and anoscopy, and graded according to the Goligher Classification (2).

Inclusion criteria were: (i) patients aged more than 18 years, (ii) hemorrhoids classified as degree II if bleeding, III, or IV if simple irreducible without ischemic changes, (iii) follow-up of at least 6 months (June 2022), (iv) failure to conservative or anal sparing treatments. Colonoscopy was performed to rule out inflammatory bowel disease, undiagnosed anal intraepithelial neoplasia, anal cancer, or other colorectal disease in patients with suspected symptoms or indications for screening (20, 21).

Exclusion criteria were: (i) fixed, fibrotic piles, degree IV hemorrhoids at advanced stage (irreducible hemorrhoids with ischemic changes and/or thrombosed), (ii) anorectal sepsis, (iii) hemorrhoids responsive to conservative treatments, (iv) previous anorectal surgery and/or anorectal cancer, (v) concomitant rectocele.

After enrolment (T0), all patients were outpatient-evaluated at 1 week (T1), 2 weeks (T2) and 1 month (T3) after surgery. Then, the follow-up was carried out with a telephone interview 6 months after the procedure (T4) (22, 23).

Subjective evaluations were obtained with the visual analog scale for pain (VAS) scores: 0 if “no pain” to 10 points if “worst imaginable pain”. All post-operative complications were graded according to Clavien–Dindo Classification (CDC) (24). Recurrences were defined as a re-bleeding in case of degree II HD or re-bleeding with prolapse in case of degree III-IV HD, recorded during follow-up outpatient visits. Rectal tenesmus was defined as the feeling of being unable to empty the large bowel, even if there is no remaining stool to expel. Anal continence was evaluated at post-operative 1 week, 1 month and 6 months using Vaizey incontinence score (23). Vaizey score, based on the Wexner score which cross-tabulates frequencies and different anal incontinence presentations, adds the use of constipating medication and the presence of fecal urgency, and ranged from 0 to 24. Hemorrhoid Severity Score (HSS), ranging from 4 to 20, was used both at the baseline, to quantify symptoms severity, and in post-treatment patient follow-up, to grade the response to treatment (22). The total HSS is obtained by the sum of the “PNR-Bleed” (more details in Appendix).

The study was conducted in accordance with the Declaration of Helsinki (1996) and International Conference on Harmonization-Good Clinical Practice guidelines (25). Internal Ethical Committee approved the study. Written informed consent was obtained from all the patients included in the study.

### Surgical technique

The patient underwent general or spinal anesthesia. The procedure was performed (12) using the THD Doppler Kit (THD Slide® S.p.A., Correggio., Italy) (12). A prophylactic dose of cephazolin antibiotic was administered only pre-operatively. The patient was positioned in a lithotomy position. The surgeon precisely located terminal branches of the rectal arterial vessels, using Doppler ultrasonography (DGHAL), and ligated them, reducing excessive blood flow to hemorrhoid cushions. Thus, the surgeon repeated the procedure moving clockwise. If hemorrhoids were prolapsed outside the anus, the mucopexy aimed to reposition the hemorrhoidal mucosa in its anatomical position. A recommended oral dose of ketorolac tromethamine of 10 mg every 8 h, not exceeding 30 mg per day, was administered during the first 24 h after surgery. Moreover, patients were encouraged to prevent hard stool by taking stool softeners as well as a high-fiber intake diet during the first 30 post-operative days. Flebotonics were associated during the same latter period.

### Statistical analysis

Descriptive statistics were reported as means ± Standard Deviation (SD) when normally distributed, and as median and interquartile range (IQR) if not normally distributed. Chi-squared test was used; a *p*-value < 0.05 was considered statistically significant. All statistical analyses were performed using the SPSS version 26.0 software package (IBM Corp., Chicago, IL, USA).

## Results

### Patients

During the period of January 2017 and December 2021, a total of 120 patients underwent a non-conservative treatment for hemorrhoids. Overall, the Milligan-Morgan hemorrhoidectomy was performed in 33 patients (24 with degree III, 7 with degree IV non-circumferential thrombosed, 2 with circumferential thrombosed hemorrhoids), the Ferguson procedures in 8 patients with degree IV with ischemic changes, and the stapled hemorrhoidectomy in 4 patients with concomitant rectocele.

The present study included 75 (62.5%) non-consecutive patients underwent THD for HD degree II (*n* = 25, 33.3%), III (*n* = 40, 53.4%) and IV (*n* = 10, 13.3%). Most were males (62.7%), with a mean age of 50 years. Preoperative median HSS was 12 (7–16). The demographic and clinicopathology features were summarized in Table 1.

**Table 1:** Patients’ characteristics. SD, standard deviation; HSS, hemorrhoid severity score; IQR, interquartile range.

Patients (*n*)	75
Female/male (*n*, %)	28 (37.3%)/47 (62.7%)
Age (±SD)	50 ± 17,92
Haemorrhoidal degree (*n*)	II = 25 (33.3%)
	III = 40 (53.4%)
	IV = 10 (13.3%)
HSS (IQR)	12 (7–16)

### In-hospital outcomes (T0)

The median time for the actual surgical treatment was 35 min. No intraoperative complications occurred (Table 2). All procedures were carried out in Day Surgery regimen with a median length-of-stay of 1 day: particularly, 73 patients (97.3%) were discharged in post-operative day 1, and 2 patients (2.7%) in day 2.

**Table 2:** Procedural results (T0), number (percentage). IQR, interquartile range.

Epidural anesthesia/General anesthesia (*n*, %)	60 (80%)/15 (20%)
Median operative time (min) (IQR)	35 (28–60)
Hospital stay (days) (IQR)	1 (0–2)

Urinary retention happened in about 21.3% of cases limited to post-operative day 1 (16 patients). Only 1 patient (1.3%) experienced persistent bleeding soon after the procedure.

### Post-operative outcomes (T1–T4)

Post-operative outcomes were classified in Table 3. Median length of registered follow-up in our cohort was 9 (6–15) months. Twenty-four patients (32%) referred at least one episode of soiling, 6 (8%) occasionally bleeding, and 37 (44%) itching during the first post-operative week (T1). There were 22 (29.3%) patients with rectal tenesmus at T1; 6 patients (8%) experienced tenesmus for a longer time (T4), including 4 with degree III and 2 with degree IV (*p* = 0.133). Overall, 28 patients felt pain at T1 with a median VAS of 6 (0–8); 96.4% (27/28) of patients had no more pain at T3. Daily routines were resumed immediately by all our patients and each of them returned to their usual professional activity within one week, with no impairment. Complete remission of symptoms occurred in 65/75 (86.7%) patients within T4.

**Table 3:** Postoperative complications (T1–T4): 1 week (T1), 2 weeks (T2), 1 month (T3), 6 months (T4) after surgery. VAS, visual analog scale; HSS, hemorrhoid severity score; IQR, interquartile range.

	T1	T2	T3	T4
Soiling (*n*, %)	24 (32%)	10 (13.3%)	4 (5.3%)	4 (5.3%)
Bleeding (*n*, %)	6 (8%)	1 (1.3%)	0	0
Itching (*n*, %)	37 (44%)	21 (28%)	13 (17.3%)	0
Tenesmus (*n*, %)	22 (29.3%)	19 (25.3%)	12 (16%)	6 (8%)
Pain (*n*, %), VAS (IQR)	28 (37.3%), 6 (0–8)	9 (12%), 4 (0–6)	1 (1.3%), 5	0
Recurrence (*n*, %)	0	0	0	2 (2.7%)*
Vaizey incontinence score (IQR)	5 (0–17)	–	3 (0–8)	0 (0–5)
HSS (IQR)	4 (4–5)	–	4	4

* Goligher's degree III.

Median Vaizey incontinence score and HSS were showed in Table 3. Five patients (6.7%) experienced faecal urgency, alteration in lifestyle and/or the need to take antidiarrheal medications in the first post-operative week (T1). No patients referred anal incontinence at 6 months (T4) after surgery. No other kind of severe complications (CDC > 2) occurred.

Recurrences were registered in 2 patients with first HD degree III (2.7%, *p* = 0.574), both experiencing re-bleeding and prolapse 6 months after surgical procedure (T4). A second THD procedure was performed.

## Discussion

Hemorrhoidal disease affects 50% of the over-50 people worldwide (1). Etiology is complex and not fully understood. In many cases, hemorrhoids are associated with conditions that increase pressure in the hemorrhoidal venous plexus, such as straining during bowel movements secondary to constipation. Other associations include obesity, pregnancy, chronic diarrhea, anal intercourse, cirrhosis with ascites, pelvic floor dysfunction, and a low-fiber diet (26).

The increase in prevalence in developed countries led to the need to organize fast-track procedures, with short operative times, very early discharge, and rapid return to work activities (27). Anyway, when conservative treatment fails, consisting of a diet rich in fiber, lactulose, and flavonoid mixture (diosmin, troxerutin, rutin, hesperidin, quercetin) (28), surgery is a feasible and suitable option, optimally improving the patient's QoL (9). Debate continues about the best surgical technique of management of mild-severe HD (12–14). Recent literature demonstrates that when compared to conventional hemorrhoidectomy, modern non-invasive surgical procedures for internal hemorrhoids, such as THD, reduce postoperative pain and facilitate a quicker discharge (7, 29). Indeed, although the “true” etiopathogenesis is still debated (mucosal prolapse (30) or “vascular hypothesis”), the THD technique would treat both causes. To date, the use of Doppler transducer is controversial (31). Nonetheless, while effective DGHAL reduces vascular flow to the hemorrhoid pads, mucopexy resolves the prolapse, resulting in THD being safe and effective in both primary and recurrent hemorrhoids (27).

The rationale of the potential clinical benefit of THD in HD is based upon three main cornerstones:

1. Therapeutic alternative in non-eligible patients. After rubber band ligation of hemorrhoids, secondary bleeding normally occurs in 10 to 14 days and patients taking anti-platelet and/or anticoagulant medication may have a higher risk, with some reports of massive life-threatening hemorrhage (32). However, Hite et al. reported that the risk of bleeding complication does not appear to be increased in patients taking clopidogrel (33). In 2016, Atallah et al reported similar rate of postoperative morbidity and hemorrhage between anticoagulated patients and who were not taking anticoagulant therapy (34), proving the safety of THD.
2. Low incidence of post-operative pain as well as other complications, and potential improvement in QoL. It is well known that THD technique is effective and safe for all degrees of hemorrhoids because of minor postoperative pain and low post-operative complication rate (7, 9, 35–37). Pain following THD was referred by up to 35% of operated patients. Yet, in most series, the incidence of postoperative pain was less than 10% (35). Postoperative bleeding was described up to 13% of patients and, in rare instances, required hospital admission and reintervention. A 2015 large meta-analysis (including 98 trials, 7827 participants, 11 surgical treatments for degree III- IV HD) suggested that THD had significantly less postoperative bleeding than other procedures and resulted in significantly fewer emergency reoperations (7). When compared with stapled hemorrhoidectomy, THD has similar early postoperative complications, but lower postoperative pain and, globally, greater patient satisfaction (38–40). Other postoperative events include tenesmus, which is more frequent in patients who underwent mucopexy, hemorrhoidal thrombosis (8.6%) and anal fissure (0.6–1.5%). Transient fecal urgency has been also reported (9, 13). Additionally, patients returned to normal daily activities (7) and work earlier compared to patients who underwent stapled hemorrhoidectomy (36). Finally, pain resolution and no postoperative constipation at 1–6 months after surgery result in high satisfaction and improved QoL after surgery (41). Despite the QoL should be a main endpoint (42), there are not hemorrhoid specific QoL score. A study using SF-36 score showed that, in addition to a reduction of symptoms (bleeding, painful defecation, anal pain, constipation and tenesmus), QoL was improved 1-month after THD: patients had reduced limitations in usual daily and social activities through increased vitality and energy, reduced psychologic distress and well-being, and decreased physical and emotional problems (43). Ain et al. described that only 12.5% of patients were not satisfied with the procedure, most of them affected by recurrence. Interestingly, there was no correlation with gender, age, constipation, Goligher Classification or other symptoms (44).

3. Reduction of recurrence and reoperation. Although THD is a non-invasive and safe procedure with lower rate of postoperative bleeding and fewer emergency reoperations compared with other procedures (7), many trials described a significant recurrence rate compared to stapled hemorrhoidectomy (38, 40). In a 2018 meta-analysis on 1,077 patients, stapled hemorrhoidectomy and THD showed comparable postoperative morbidity, while the former seemed to have lower recurrence rate (38). Similarly, a recent study on 554 patients described persistent or recurrent HD in 13.2% and 6.9% patients after THD and stapled hemorrhoidectomy, respectively (40). Negative prognostic factors were younger age, degree IV disease, and high artery ligation (10).

Overall, the 2020 Practice Parameters for Management of Hemorrhoids (45) recommend THD in high- degree (II and III) hemorrhoids (2) and/or after medical therapy failure. No unanimous agreement has been reached regarding the efficacy and safety in degree IV hemorrhoids. Sobrado et al. emphasized that, due to its high rate of prolapse and bleeding, THD is not an effective option for the treatment of degree IV hemorrhoids (46). Genova et al. showed that Milligan-Morgan hemorrhoidectomy had similar clinical outcomes in degree III HD and better results in degree IV HD when compared with THD (47). Moreover, Ratto and Giordano suggested THD with mucopexy when symptoms are mostly transient, occasional, or limited in severity (48). A review of 28 prospective studies, including 2,904 patients with grade I to IV hemorrhoids, described a recurrence rate of 3–60%, with the highest for grade IV hemorrhoids. Therefore, only hemorrhoids classified as degree IV at initial stage were included in our series, while fixed, fibrotic piles, necrotic advanced hemorrhoids were treated first with excisional hemorrhoidectomy (Milligan-Morgan and Ferguson procedures). Despite the limited number of patients (*n* = 10, 13.3%), no one complained of disease recurrence (46–48).

In our series, we focused on four different points in time post-operatively: at 1 week = T1, at 2 weeks = T2, at 1 month = T3, and at 6 months = T4. We evaluated the occurrence of symptoms such as soiling, bleeding, itching, tenesmus, and pain as well as disease recurrences. Moreover, we measured the Vaizey incontinence score and the HSS at T1, T3 and T4. Overall, we found out that our patients moderately experienced bleeding (8% of patients at T1 and 1.3% at T2) and pain (37.3% of patients at T1% and 12% at T2), which decreased dramatically in the following controls. At T4 most of the symptoms were completely gone except for some patients who still experienced soiling (5.3%) and tenesmus (8%), thus documenting the absence of severe complications, such as bleeding and pain at T4. The novel Anolift technique may allow for a more even distribution of the tension along the suture lines and reduce the risks of creating a pocket in the rectal lumen, resulting in a lower rate of persistent rectal tenesmus (up to 1 in 10 patients at 6 months after surgery). Moreover, median Vaizey incontinence score decreased until reaching the minimum score at T4. Post-operative HSS highlights the efficacy of THD, which definitively results in improved QoL of patients. Lastly, recurrence rate was surprisingly low with only 2 cases (2.7%), probably as a result of a limited postoperative follow-up.

Interestingly, in our previous experience, neither 30-days severe postoperative complications nor postoperative readmission were registered; tenesmus occurred in 75% of patients underwent THD for degree II and III, which, however, solved spontaneously on the first postoperative day (27). Even though other studies had much higher number of patients to work on, the results of our study were somewhat similar to those of Ratto et al. showed even better outcomes in bleeding and pain 1 month after surgery and in recurrence rate (10). In the present study, THD has a low rate of symptom relapse and recurrence even in stage IV disease. We argue that high recurrence rate following THD, as reported in previous literature, could be influenced by technical experience (49).

Nowadays, the treatment of HD constitutes a narrow-minded approach that doesn't account for patients' needs, expectations and personal characteristics often leading to a blurry definition for success of surgical procedures in the long term and not compelling for an approach tailored to every single patient (50–52). The surgeon's experience seems to be the only key factor in the decision on surgical technique. Consequently, it remains unclear how much the surgeon's skill affects the outcome of patients (8).

The THD is a safe and effective atraumatic technique associated, not influencing sphincter complex or anal function, with the best short-term clinical and surgical outcomes (rapid symptoms relief, lesser surgical site infection (53) and postoperative complications). Fast postoperative recovery, early discharge, and quickly return to normal daily activities and works substantially improves patient's QoL (54).

The main limitation of our observational study is a small cohort of enrolled patients, even given that all procedures were performed by the same expert surgeon, and a limited follow-up. Despite its exploratory nature, our study offers some insight into the “real” clinical practice. Further prospective studies are necessary to implement the paucity of evidence still available, investigating, on one hand, long-term outcomes, QoL and patients' satisfaction, and, on the other hand, predictive factors of recurrence.

## Conclusion

THD is a safe and effective procedure for selected patients with hemorrhoids of every degree, with no significant differences in the rates of post-operative complications or recurrences, and improved patient's QoL. We recommend THD as a valid therapeutic option for Goligher's degree II and III hemorrhoids.

## Data Availability

The raw data supporting the conclusions of this article will be made available by the authors, without undue reservation.

## Appendix

The HSS is the total score obtained by the sum of the numerical grades of all four characteristics of hemorrhoids in “PNR-Bleed” classification:

• Degree of hemorrhoidal Prolapse (P): 1 point for no hemorrhoidal prolapse (Goligher's degree I), 2 prolapse upon straining that reduces spontaneously (Goligher's degree II), 3 prolapse upon straining that needs manual reduction (Goligher's degree III), 4 prolapsed and irreducible hemorrhoids but without ischemic changes (Goligher's degree IV), 5 prolapsed and irreducible hemorrhoids with ischemic (gangrenous) changes (Goligher's degree IV).
• Number of hemorrhoidal columns involved (*N*): 1 point for one column, 2 two, 3 three, 4 four, 5 circumferential (presence of secondary hemorrhoids along with the involvement of all primary hemorrhoids).
• Relation of the hemorrhoidal tissue to dentate line (R): 1 point for nil (normal anal cushions), 2 external hemorrhoids, 3 internal hemorrhoids, 4 interno-external hemorrhoids, 5 thrombosed external hemorrhoids.
• Bleeding: 1 point for nil, 2 mild—occasional episodes (during defecation), 3 moderate—frequent episodes (during defecation), 4 severe—persistent bleeding even without defecation with fall in Hb level (<10 gm/dl), requiring hematinics, 5 very severe—bleeding in the form of jets and splashes with severe fall in Hb level (<7 gm/dl), requiring blood transfusion.

## References

[1] Gallo G Sacco R Sammarco G. Hemorrhoids coloproctology. Cham: Springer (2018). 3–7 p. 10.1007/978-3-319-53357-5_1

[2] Goligher J Duthie H Nixon H. Surgery of the anus, rectum and colon. 5th ed. London: Balliere Tindall (1984).

[3] Stratta E Gallo G Trompetto M. Conservative treatment of hemorrhoidal disease. Rev Recent Clin Trials. (2021) 16:87–90. 10.2174/1574887115666201021150144 33087033

[4] Cocorullo G Tutino R Falco N Licari L Orlando G Fontana T The non-surgical management for hemorrhoidal disease. A systematic review. II Giornale di Chirurgia/J Surg. (2017) 38:5–14. 10.11138/gchir/2017.38.1.005 PMC5730401 28460197

[5] Cengiz TB Gorgun E. Hemorrhoids: a range of treatments. Cleve Clin J Med. (2019) 86:612–20. 10.3949/ccjm.86a.18079 31498764

[6] Dekker L Han-Geurts IJM Grossi U Gallo G Veldkamp R. Is the goligher classification a valid tool in clinical practice and research for hemorrhoidal disease? Tech Coloproctol. (2022) 26:387–92. 10.1007/s10151-022-02591-3 35141793 PMC9018630

[7] Simillis C Thoukididou SN Slesser AAP Rasheed S Tan E Tekkis PP. Systematic review and network meta-analysis comparing clinical outcomes and effectiveness of surgical treatments for haemorrhoids. Br J Surg. (2015) 102:1603–18. 10.1002/bjs.9913 26420725

[8] Morinaga K Hasuda K Ikeda T. A novel therapy for internal hemorrhoids: ligation of the hemorrhoidal artery with a newly devised instrument (moricorn) in conjunction with a Doppler flowmeter. Am J Gastroenterol. (1995) 90:610–3. 7717320

[9] Gallo G Martellucci J Sturiale A Clerico G Milito G Marino F Consensus statement of the Italian society of colorectal surgery (SICCR): management and treatment of hemorrhoidal disease. Tech Coloproctol. (2020) 24:145–64. 10.1007/s10151-020-02149-1 31993837 PMC7005095

[10] Ratto C Campennì P Papeo F Donisi L Litta F Parello A. Transanal hemorrhoidal dearterialization (THD) for hemorrhoidal disease: a single-center study on 1000 consecutive cases and a review of the literature. Tech Coloproctol. (2017) 21:953–62. 10.1007/s10151-017-1726-5 29170839 PMC5830492

[11] Pucher PH Sodergren MH Lord AC Darzi A Ziprin P. Clinical outcome following Doppler-guided haemorrhoidal artery ligation: a systematic review. Colorectal Dis. (2013) 15:e284–94. 10.1111/codi.12205 23489678

[12] Ratto C. THD Doppler procedure for hemorrhoids: the surgical technique. Tech Coloproctol. (2014) 18:291–8. 10.1007/s10151-013-1062-3 24026315 PMC3950605

[13] Giordano P Tomasi I Pascariello A Mills E Elahi S. Transanal dearterialization with targeted mucopexy is effective for advanced haemorrhoids. Colorectal Dis. (2014) 16:373–6. 10.1111/codi.12574 24460621 PMC4283720

[14] Giordano P Schembari E. Transanal hemorrhoidal dearterialization (THD) anolift-prospective assessment of safety and efficacy. Front Surg. (2021) 8:373–6. 10.3389/fsurg.2021.704164 34631778 PMC8493063

[15] Aibuedefe B Kling SM Philp MM Ross HM Poggio JL. An update on surgical treatment of hemorrhoidal disease: a systematic review and meta-analysis. Int J Colorectal Dis. (2021) 36:2041–9. 10.1007/s00384-021-03953-3 34101003

[16] Davis BR Lee-Kong SA Migaly J Feingold DL Steele SR. The American society of colon and rectal surgeons clinical practice guidelines for the management of hemorrhoids. Dis Colon Rectum. (2018) 61:284–92. 10.1097/DCR.0000000000001030 29420423

[17] Trompetto M Clerico G Cocorullo GF Giordano P Marino F Martellucci J Evaluation and management of hemorrhoids: italian society of colorectal surgery (SICCR) consensus statement. Tech Coloproctol. (2015) 19:567–75. 10.1007/s10151-015-1371-9 26403234

[18] Guyatt G Gutterman D Baumann MH Addrizzo-Harris D Hylek EM Phillips B Grading strength of recommendations and quality of evidence in clinical guidelines. Chest. (2006) 129:174–81. 10.1378/chest.129.1.174 16424429

[19] Von EE Altman D Egger M. The strengthening the reporting of observational studies in epidemiology(STROBE) statement: guidelines for reporting observational studies. Prev Med. (2007) 45:247–51. 10.1016/S0140-6736(07)61602-X 17950122

[20] Timaran CH Sangwan YP Solla JA. Adenocarcinoma in a hemorrhoidectomy specimen: case report and review of the literature. Am Surg. (2000) 66:789–92. 10.1177/000313480006600821 10966042

[21] Caparelli ML Batey JC Tailor A Braverman T Barrat C. Internal hemorrhoid harboring adenocarcinoma: a case report. World J Gastrointest Oncol. (2021) 13:87–91. 10.4251/wjgo.v13.i1.87 33510851 PMC7805272

[22] Khan MA Chowdri NA Parray FQ Wani RA Mehraj A Baba A “PNR-Bleed” classification and hemorrhoid severity score—a novel attempt at classifying the hemorrhoids. Ann Coloproctol. (2020) 40:398–403. 10.1016/j.jcol.2020.05.012

[23] Vaizey CJ Carapeti E Cahill JA Kamm MA. Prospective comparison of faecal incontinence grading systems. Gut. (1999) 44:77–80. 10.1136/gut.44.1.77 9862829 PMC1760067

[24] Clavien PA Barkun J de Oliveira ML Vauthey JN Dindo D Schulick RD The clavien-dindo classification of surgical complications. Ann Surg. (2009) 250:187–96. 10.1097/SLA.0b013e3181b13ca2 19638912

[25] Dixon JR. The international conference on harmonization good clinical practice guideline. Qual Assur. (1999) 6:65–74. 10.1080/105294199277860 10386329

[26] Mott T Latimer K Edwards C. Hemorrhoids: diagnosis and treatment options. Am Fam Physician. (2018) 97:172–9. 29431977

[27] Tirone A Vuolo G Gaggelli I Francioli N D’Onofrio P Quarta S Emorroidectomia con tecnica THD (transanal haemorroidal dearterialization) nostra esperienza. Ann Ital Chir. (2010) 81:311–3. 21322276

[28] Corsale I Carrieri P Martellucci J Piccolomini A Verre L Rigutini M Flavonoid mixture (diosmin, troxerutin, rutin, hesperidin, quercetin) in the treatment of I–III degree hemorroidal disease: a double-blind multicenter prospective comparative study. Int J Colorectal Dis. (2018) 33:1595–600. 10.1007/s00384-018-3102-y 29934701

[29] Kline RP. Operative management of internal hemorrhoids. J Am Acad Physician Assist. (2015) 28:27–31. 10.1097/01.JAA.0000459809.87889.85 25594292

[30] Thomson WHF. The nature of haemorrhoids. Br J Surg. (2005) 62:542–52. 10.1002/bjs.1800620710 1174785

[31] Schuurman J-P Rinkes IHMB Go PMNYH. Hemorrhoidal artery ligation procedure with or without Doppler transducer in grade II and III hemorrhoidal disease. Ann Surg. (2012) 255:840–5. 10.1097/SLA.0b013e31824e2bb5 22504188

[32] Albuquerque A. Rubber band ligation of hemorrhoids: a guide for complications. World J Gastrointest Surg. (2016) 8:614–20. 10.4240/wjgs.v8.i9.614 27721924 PMC5037334

[33] Hite N Klinger AL Miller P Beck DE Whitlow CB Hicks TC Clopidogrel bisulfate (plavix) does not increase bleeding complications in patients undergoing rubber band ligation for symptomatic hemorrhoids. J Surg Res. (2018) 229:230–3. 10.1016/j.jss.2018.04.004 29936995

[34] Atallah S Maharaja GK Martin-Perez B Burke JP Albert MR Larach SW. Transanal hemorrhoidal dearterialization (THD): a safe procedure for the anticoagulated patient? Tech Coloproctol. (2016) 20:461–6. 10.1007/s10151-016-1481-z 27170327 PMC4920854

[35] Dal Monte PP Tagariello C Sarago M Giordano P Shafi A Cudazzo E Transanal haemorrhoidal dearterialisation: nonexcisional surgery for the treatment of haemorrhoidal disease. Tech Coloproctol. (2007) 11:333–8: discussion 338–9. 10.1007/s10151-007-0376-4 18060529

[36] Giordano P Nastro P Davies A Gravante G. Prospective evaluation of stapled haemorrhoidopexy versus transanal haemorrhoidal dearterialisation for stage II and III haemorrhoids: three-year outcomes. Tech Coloproctol. (2011) 15:67–73. 10.1007/s10151-010-0667-z 21318581 PMC3046344

[37] Tsang YP Fok KLB Cheung YSH Li KWM Tang CN. Comparison of transanal haemorrhoidal dearterialisation and stapled haemorrhoidopexy in management of haemorrhoidal disease: a retrospective study and literature review. Tech Coloproctol. (2014) 18:1017–22. 10.1007/s10151-014-1170-8 24906978

[38] Xu L Chen H Gu Y. Stapled hemorrhoidectomy versus transanal hemorrhoidal dearterialization in the treatment of hemorrhoids: an updated meta-analysis. Surg Laparosc Endosc Percutan Tech. (2019) 29:75–81. 10.1097/SLE.0000000000000612 30540639

[39] Song Y Chen H Yang F Zeng Y He Y Huang H. Transanal hemorrhoidal dearterialization versus stapled hemorrhoidectomy in the treatment of hemorrhoids: a PRISMA-compliant updated meta-analysis of randomized control trials. Medicine (Baltimore). (2018) 97:e11502. 10.1097/MD.0000000000011502 30024532 PMC6086545

[40] Emile SH Elfeki H Sakr A Shalaby M. Transanal hemorrhoidal dearterialization (THD) versus stapled hemorrhoidopexy (SH) in treatment of internal hemorrhoids: a systematic review and meta-analysis of randomized clinical trials. Int J Colorectal Dis. (2019) 34:1–11. 10.1007/s00384-018-3187-3 30421308

[41] Zampieri N Castellani R Andreoli R Geccherle A. Long-term results and quality of life in patients treated with hemorrhoidectomy using two different techniques: ligasure versus transanal hemorrhoidal dearterialization. Am J Surg. (2012) 204:684–8. 10.1016/j.amjsurg.2012.01.014 23140829

[42] Johannsson HO Graf W Pahlman L. Bowel habits in hemorrhoid patients and Normal subjects. Am J Gastroenterol. (2005) 100:401–6. 10.1111/j.1572-0241.2005.40195.x 15667500

[43] Talha S Burke JP Waldron D Coffey JC Condon E. Early quality of life outcomes following Doppler guided transanal haemorrhoidal dearterialisation: a prospective observational study. Acta Gastroenterol Belg. (2013) 76:231–4. 23898561

[44] Ain Q U Bashir Y Eguare E. Evaluation of the effectiveness and patients’ contentment with transanal haemorrhoidal artery dearterialisation and mucopexy (THD) for treatment of haemorrhoidal disease: a 6-year study. Ir J Med Sci. (2018) 187:647–55. 10.1007/s11845-017-1715-8 29214383

[45] Rivadeneira DE Steele SR Ternent C Chalasani S Buie WD Rafferty JL. Practice parameters for the management of hemorrhoids (revised 2010). Dis Colon Rectum. (2011) 54:1059–64. 10.1097/DCR.0b013e318225513d 21825884

[46] Sobrado CW Klajner S Hora JAB Mello A da Silva FML Frugis MO Transanal haemorrhoidal dearterialization with mucopexy (THD-M) for treatment of hemorrhoids: is it applicable in all grades? Brazilian multicenter study. Arq Bras Cir Dig. (2020) 33:e1504. 10.1590/0102-672020190001e1504 32844877 PMC7448859

[47] Genova P Damiano G lo Monte AI Genova G. Transanal hemorrhoidal dearterialization versus milligan-morgan hemorrhoidectomy in grade III/IV hemorrhoids. Ann Ital Chir. (2019) 90:145–51. 31182699

[48] Ratto C Giordano P Donisi L Parello A Litta F Doglietto GB. Transanal haemorrhoidal dearterialization (THD) for selected fourth-degree haemorrhoids. Tech Coloproctol. (2011) 15:191–7. 10.1007/s10151-011-0689-1 21505901

[49] Popov V Yonkov A Arabadzhieva E Zhivkov E Bonev S Bulanov D Doppler-guided transanal hemorrhoidal dearterilization versus conventional hemorrhoidectomy for treatment of hemorrhoids – early and long-term postoperative results. BMC Surg. (2019) 19(4):4. 10.1186/s12893-019-0469-9 30630463 PMC6327383

[50] Gallo G Pietroletti R Novelli E Sturiale A Tutino R Lobascio P A multicentre, open-label, single-arm phase II trial of the efficacy and safety of sclerotherapy using 3% polidocanol foam to treat second-degree haemorrhoids (SCLEROFOAM). Tech Coloproctol. (2022) 26:627–36. 10.1007/s10151-022-02609-w 35334004 PMC8949823

[51] Gallo G Picciarielllo A Pietroletti R Novelli E Sturiale A Tutino R Sclerotherapy with 3% polidocanol foam to treat second-degree haemorrhoidal disease: 3-year follow-up of a multicentre, single arm, IDEAL phase 2b trial. Colorectal Dis. (2022) 1–10. 10.1111/codi.16380 36268758

[52] Brown SR Tiernan JP Watson AJM Biggs K Shephard N Wailoo AJ Haemorrhoidal artery ligation versus rubber band ligation for the management of symptomatic second-degree and third-degree haemorrhoids (HubBLe): a multicentre, open-label, randomised controlled trial. Lancet. (2016) 388:356–64. 10.1016/S0140-6736(16)30584-0 27236344 PMC4956910

[53] Marano L Carbone L Poto GE Calomino N Neri A Piagnerelli R Antimicrobial prophylaxis reduces the rate of surgical site infection in upper gastrointestinal surgery: a systematic review. Antibiotics. (2022) 11:230. 10.3390/antibiotics11020230 35203832 PMC8868284

[54] Li D Jensen C. Patient satisfaction and quality of life with enhanced recovery protocols. Clin Colon Rectal Surg. (2019) 32:138–44. 10.1055/s-0038-1676480 30833864 PMC6395092

